# Two repetition time saturation transfer (TwiST) with spill-over correction to measure creatine kinase reaction rates in human hearts

**DOI:** 10.1186/s12968-015-0175-4

**Published:** 2015-08-08

**Authors:** Michael Schär, Refaat E. Gabr, AbdEl-Monem M. El-Sharkawy, Angela Steinberg, Paul A. Bottomley, Robert G. Weiss

**Affiliations:** Division of MR Research, Russell H. Morgan Department of Radiology and Radiological Science, The Johns Hopkins University School of Medicine, Baltimore, MD USA; Department of Diagnostic and Interventional Imaging, University of Texas Health Science Center at Houston, Houston, TX USA; Systems and Biomedical Engineering Department, Faculty of Engineering, Cairo University, Giza, Egypt; Cardiology Division, Department of Medicine, The Johns Hopkins University School of Medicine, Baltimore, MD USA

**Keywords:** Saturation transfer, Human heart, Metabolism, Reaction rate, High-energy phosphate, Heart failure, 3 Tesla, Creatine-kinase, TwiST

## Abstract

**Background:**

Phosphorus saturation transfer (ST) magnetic resonance spectroscopy can measure the rate of ATP generated from phosphocreatine (PCr) via creatine kinase (CK) in the human heart. Recently, the triple-repetition time ST (TRiST) method was introduced to measure the CK pseudo-first-order rate constant k_f_ in three acquisitions. In TRiST, the longitudinal relaxation time of PCr while γ-ATP is saturated, T_1_`, is measured for each subject, but suffers from low SNR because the PCr signal is reduced due to exchange with saturated γ-ATP, and the short repetition time of one of the acquisitions. Here, a two-repetition time ST (TwiST) method is presented. In TwiST, the acquisition with γ-ATP saturation and short repetition time is dropped. Instead of measuring T_1_`, an intrinsic relaxation time T_1_ for PCr, T_1_^intrinsic^, is assumed. The objective was to validate TwiST measurements of CK kinetics in healthy subjects and patients with heart failure (HF).

**Methods:**

Bloch equation simulations that included the effect of spillover irradiation on PCr were used to derive formulae for T_1_^intrinsic^ and k_f_ measured by both TRiST and TwiST methods. Spillover was quantified from an unsaturated PCr measurement used in the current protocol for determining PCr and ATP concentrations. Cardiac TRiST and TwiST data were acquired at 3 T from 12 healthy and 17 HF patients.

**Results:**

Simulations showed that both k_f_ measured by TwiST and T_1_^intrinsic^ require spill-over corrections. In human heart at 3 T, the spill-over corrected T_1_^intrinsic^ = 8.4 ± 1.4 s (mean ± SD) independent of study group. TwiST and TRiST k_f_ measurements were the same, but TwiST was 9 min faster. Spill-over corrected TwiST k_f_ was 0.33 ± 0.08 s^−1^ vs. 0.20 ± 0.06 s^−1^ in healthy vs HF hearts, respectively (*p* < 0.0001).

**Conclusion:**

TwiST was validated against TRiST in the human heart at 3 T, generating the same results 9 min faster. TwiST detected significant reductions in CK k_f_ in HF compared to healthy subjects, consistent with prior 1.5 T studies using different methodology.

**Electronic supplementary material:**

The online version of this article (doi:10.1186/s12968-015-0175-4) contains supplementary material, which is available to authorized users.

## Background

Phosphorus (^31^P) saturation transfer (ST) magnetic resonance spectroscopy (MRS) enables the *in vivo* study of adenosine triphosphate (ATP) kinetics including those through the creatine kinase (CK) reaction [1, 2]. In muscle, the CK reaction serves as the prime energy reserve and a putative shuttle, transporting high-energy phosphates between the mitochondria, where ATP is created, and the myofibrils, where it is used. The pseudo-first-order rate constant (k_f_) for CK indexes the fractional rate of ATP generation from phosphocreatine (PCr). ^31^P ST MRS using the Four Angle Saturation Transfer (FAST; [3]) protocol at 1.5 T enabled human cardiac CK kinetic studies for the first time and identified significant reductions in cardiac k_f_ in patients with heart failure (HF). These findings and others indicated that reduced CK energy supply occurs in human HF, may play a role in the associated contractile dysfunction [4, 5], and is an independent predictor of subsequent clinical HF events [6].

In a ST experiment, the pseudo-first-order rate constant can be determined with:

1 $$ {k}_f=\frac{1}{T_1\hbox{'}}\left(1-\frac{M_0\hbox{'}}{M_0}\right), $$

where T_1_` and M_0_` are the longitudinal relaxation time and the equilibrium magnetization of PCr measured while the exchanging γ-ATP resonance at −2.5 ppm is selectively saturated (signified by primes); and M_0_ is the equilibrium magnetization of PCr measured without γ-ATP saturation. Recently, the Triple Repetition time ST (TRiST) protocol was introduced to more efficiently measure k_f_ in the human heart at 3 T [7]. TRiST only requires three acquisitions to determine k_f_. Each of the three acquisitions takes between 8 and 22 min as they employ chemical shift imaging (CSI) for localization and multiple averages for sufficient signal-to-noise ratio (SNR). Using TRiST, T_1_` and M_0_` are measured by the dual-repetition time (TR) method [8] with a short TR (M`(TR_short_); TR_short_ = 2 heart beats, cardiac-gated) and a long TR (M`(TR_long_); TR_long_ ~10 s, cardiac-gated) both while the exchanging γ-ATP resonance is saturated. A third cardiac-gated acquisition at a TR of ~ 16 s is performed to measure M_0_. To compensate for the effects of spill-over irradiation on PCr during γ-ATP saturation, this third acquisition is performed while applying saturation at +2.5 ppm–termed “control saturation”, yielding M_0_^control^. Equation [1] is then written as:

2 $$ {k}_f^{TRiST}=\frac{1}{T_1\hbox{'}}\left(1-\frac{M_0\hbox{'}}{M_0^{control}}\right). $$

Spill-over irradiation is caused by imperfect frequency-selective saturation of γ-ATP that can partially saturate the nearby PCr resonance. A measure of the spill-over effect is provided by the ratio

3 $$ Q=\frac{M_0^{control}}{M_0}, $$

of the PCr signal acquired with control saturation, to the PCr signal acquired without any saturation [3, 9]. The control saturation experiment does not fully compensate for the effect of spill-over on the observed k_f_, and several methods have been presented to correct for the residual errors [9, 10]. Nevertheless, the rate constant measured with TRiST appears relatively robust to varying levels of spill-over, as evidenced, for example, by essentially constant leg k_f_ measurements at 3 T over regions wherein Q varied from 0.5 to 0.9 [7].

The goal of the present work is to introduce and validate an even more efficient two-repetition time ST (TwiST) method for measuring k_f_. TwiST is based on prior knowledge of the so-called “intrinsic T_1_” of PCr (T_1_^intrinsic^) which is independent of the chemical exchange processes implicit in Eqs [1] and [2]. The intrinsic T_1_ was introduced long ago as the hypothetical T_1_ that would occur if there were no chemical exchange, and is given by [1, 11]:

4 $$ {T}_1^{intrinsic}={T}_1\hbox{'}\left(\frac{M_0^{control}}{M_0\hbox{'}}\right). $$

If T_1_^intrinsic^ is known and is similar among groups studied, then k_f_ is determinable from just two fully-relaxed measurements of M_0_ and M_0_` [11, 12]. These two measurements comprise the TwiST experiment. In this case, k_f_ is given by [13]:

5 $$ {k}_f^{TwiST}=\frac{1}{T_1^{intrinsic}}\left(\frac{M_0^{control}}{M_0\hbox{'}}-1\right), $$

The pre-requisite for performing TwiST is prior knowledge of T_1_^intrinsic^. Here, T_1_^intrinsic^ is determined from equation [4] and based on experimental data acquired from the hearts of healthy subjects and patients with HF. The effects of variations in the spill-over ratio, Q, on measurements of T_1_^intrinsic^, k_f_^TRiST^, and k_f_^TwiST^ are evaluated by Bloch equation analysis. Spill-over correction for T_1_^intrinsic^, k_f_^TRiST^, and k_f_^TwiST^ is derived analogous to the method described in [9]. If needed, this correction uses an unsaturated acquisition that is routinely recorded for determining PCr and ATP concentrations, and for measuring CK flux, (k_f_ x [PCr]), in standard patient protocols [4–6, 14–18].

## Methods

### Monte Carlo simulations without spill-over effects

Analogous to [7], Monte Carlo simulations were performed using Python 2.7 software (www.python.org) to determine the effect of low ^31^P SNR on k_f_^TwiST^ for 0.1 ≤ k_f_ ≤ 0.4 s^−1^ [4, 5], and 5 ≤ T_1_^intrinsic^ ≤ 9 s. These ranges were based on measured values for human heart, including an observed T_1_ of PCr, T_1,PCr_, of 5.8 s [8] which sets a lower limit to T_1_^intrinsic^ because chemical exchange with ATP reduces T_1,PCr_ [19]. Gaussian noise with a standard deviation (SD) of σ = 0.16 M_0_ per acquisition was added 2000 times to the two TWiST acquisitions, M`(TR_long_) and M_0_^control^(TR_control_). k_f_^TwiST^ was calculated from Eq. [5]. For estimating error, the SD of k_f_^TwiST^ was calculated from the 2000 runs, and then averaged for the different k_f_ and T_1_^intrinsic^. Fifty-nine different combinations of averages and cardiac-gated TRs that resulted in a fixed total scan time of ~30 min for the two acquisitions were evaluated. For each TR combination, the number of averages leading to the lowest error was selected. Bias was determined for each sequence combination with a mid-range T_1_^intrinsic^ = 7 s only.

### Bloch equation calculation of spill-over effects

In the prior TRiST protocol [7], a surface coil was used for RF transmission, producing an inhomogeneous excitation field with high intensity close to the coil and a gradual decline moving away from it. An adiabatic excitation pulse was used to generate a homogeneous flip-angle (FA) over the region of interest, and a modulated DANTE pulse train was used for saturating the exchanging γ-ATP moiety. The DANTE pulses are subject to inhomogeneity in the transmit field, and were set to provide sufficient saturation at the depth of the heart. This causes spill-over saturation of PCr closer to the coil, which is quantified by the variable Q in Eq. 3. Its effects on the measured T_1_^intrinsic^, k_f_^TRiST^ and k_f_^TwiST^ are determined here by numerical analysis of the Bloch–McConnell equations modified for two-site chemical exchange in matrix form [3, 20] implemented on a graphical programming interface (GPI) [21].

The PCr signals, M_0_(TR = 16 s), without any saturation; M_0_^control^(TR = 16 s) with control saturation at +130 Hz; and M`_PCr_(TR = 10 s) and M`_PCr_(TR = 1.7 s) with γ-ATP saturated at −130 Hz, were all determined for the TRiST and TwiST experiments. The saturation of γ-ATP by the amplitude-modulated DANTE scheme described in Eq. 5 of [7] was simulated with parameters used in human studies (m = 5 suppression bands; δ = 9 Hz separation between bands; β = 0.9° FA per band as expected 10 cm from the coil [8]; τ = 0.91 ms between hard sub-pulses of 100-μs duration).

To simulate a range of spill-over strengths, β was varied from 0.1° to 6.0°. Other parameters were: T_1_^intrinsic^ = 7900 ms; T_1,ATP_^intrinsic^ = 2200 ms for the T_1_^intrinsic^ of ATP; with corresponding spin-spin relaxation times (T_2_), T_2,PCr_ = 250 ms and T_2,ATP_ = 50 ms chosen somewhat shorter than values measured in calf muscle [22]. To simulate the effect of static magnetic field inhomogeneity, the calculations were performed 9 times with the saturation frequency offset by −20 to +20 Hz in steps of 5 Hz. Results from the 9 runs were weighted with a 20 Hz full-width-half-maximum Gaussian function and averaged. Based on the four calculated M_0_ and M` PCr signals, simulated values of T_1_^intrinsic^, k_f_^TRiST^, and k_f_^TwiST^ were determined from equations [4], [2], and [5], respectively. Two different “true” k_f_s of 0.21 s^−1^ and 0.32 s^−1^ were assumed, reflecting previous rates measured at 1.5 T for HF patients and healthy subjects, respectively [4].

### Spill-over corrections for intrinsic T_1_, TRiST and TwiST

Spill-over corrected T_1_^intrinsic^, TRiST and TwiST formulae were determined using the approach of Gabr et al. [9]. The Bloch–McConnell equations [20] were numerically solved for the range of parameters listed in Table 1, with the range of saturation power limited to Q ≤ 0.96 to ensure sufficient saturation of γ-ATP (see Discussion). A minimum sum-of-the-squared fractional differences algorithm was applied to fit the calculated data to linear spill-over corrected formulae with an affine dependence on the measured parameters. The corrected intrinsic T_1_, T_1_^Q-intrinsic^, had the form:

**Table 1:** Parameter ranges of the two exchanging metabolites of the CK reaction used to determine the spill-over corrected formulae for cardiac TRiST and TwiST at 3 Tesla

Parameter	Min	Max	Fixed
PCr/ATP	1	2	
intrinsic T_1,PCr_ [s]	6.5	9.5	
intrinsic T_1,__γ-ATP_ [s]	2	3.5	
T_2,PCr_ [s]			0.2
T_2,__γ-ATP_ [s]			0.05
k_f_ [s^−1^]	0.1	0.4	
DANTE β [°]	0.8	4.0	
Δf [Hz]			130

6 $$ {T}_1^{Q- intrinsic}=a\left(\frac{M_0\hbox{'}}{M_0^{control}}+b\right)\left(Q+c\right)\left({T}_1^{\hbox{'}}+d\right). $$

The Q-corrected TRiST, k_f_^Q-TRiST^ was:

7 $$ {k}_f^{Q- TRiST}=f\left(\frac{M_0\hbox{'}}{M_0^{control}}+g\right)\left(Q+h\right)\left({T}_1^{\hbox{'}}+k\right). $$

And the Q-corrected TwiST, k_f_^Q-TwiST^ was formulated as:

8 $$ {k}_f^{Q- TwiST}=l\left(\frac{M_0^{control}}{M\hbox{'}\left(T{R}_{long}\right)}+m\right)\left(Q+n\right), $$

where a-n are fitting coefficients.

These new spill-over corrected formulae were applied to the simulated PCr signals generated from the previous section.

### Human studies

Human studies were approved by the Institutional Review Board of the Johns Hopkins University School of Medicine, with all participants providing written informed consent. TRiST data, that included TwiST data as a subset, were acquired on a 3 T broadband Achieva scanner (Philips Healthcare, Best, the Netherlands) from twelve healthy subjects (7 men, 5 women, mean age of 36 ± 15 years) with no history of hypertension, diabetes, or heart disease; and in seventeen HF patients (8 men, 9 women, mean age of 48 ± 15 years) with a clinical history of HF (New York Heart Association class I (2), class II (8), and class III (7)), a left ventricular ejection fraction <40 %, and no significant coronary disease.

**Fig. 1:**
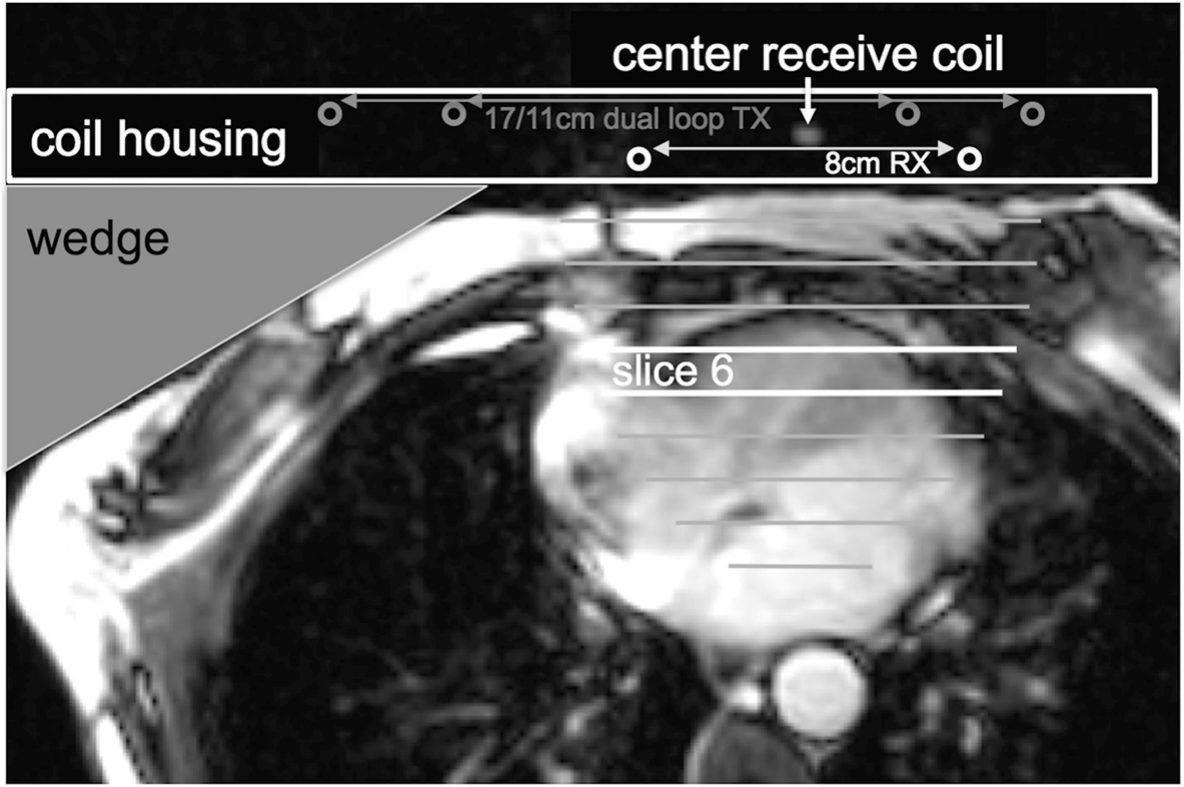
Axial, end-systolic time frame of a balanced steady-state free precession cine image illustrating the patient setup. Participants were oriented prone on the coil which includes a marker at the center of the receive coil. Circles depict location of 8 cm diameter receive (white) and 17/11 cm diameter dual loop transmit coil (gray) conductors. A wedge is used to rotate the participant to ensure the center of the receive coil is straight in front of the cardiac septum. Lines indicate the slices of 1-dimensional CSI localization combined with receive coil sensitivity. Bright lines indicate the 6^th^ slice, the origin of example spectra shown in Fig. 2

Both TwiST and reference TRiST data were acquired from a single TRiST protocol described in [7]. Guided by scout MRI, participants were oriented prone with the heart centered above a custom built ^31^P coil set with dual loop 17/11-cm diameter transmit and 8-cm receive coils [8] that had a fiducial marker at its center (Fig. 1). Localized 2^nd^-order shimming was performed based on acquired field mapping [23]. Axial balanced steady-state free precession cine images were acquired during free breathing to determine the trigger delay for MRS acquisition at end-systole, which was chosen to minimize motion and maximize the amount of cardiac tissue close to the coil. Cardiac-triggered, 1D CSI data were acquired with sixteen phase encodes from a 16-cm field-of-view using frequency-sweep-cycled adiabatic half-passage [8] excitation. A first data set was acquired without saturation at TR ≥ 16 s and 2 averages to measure M_0_. This data set was used to center the saturation frequency on the cardiac γ-ATP resonance and to determine the spill-over ratio Q; it can also be used to determine metabolite concentrations [14](not reported here). Next, three TRiST data sets were acquired: the first, M_0_^control^, with control saturation applied (TR_control_ ≥ 16 s; 2 averages); the second, M`(TR_long_), with γ-ATP saturated (TR_long_ ≥ 10 s; 8 averages); and the third, M`(TR_short_) also with γ-ATP saturated (TR_short_ = 2 heart beats; 18 averages). The third acquisition was not used in the TwiST analysis. The average TR of each triggered acquisition was determined from the scanner’s physiological log.

**Fig. 2:**
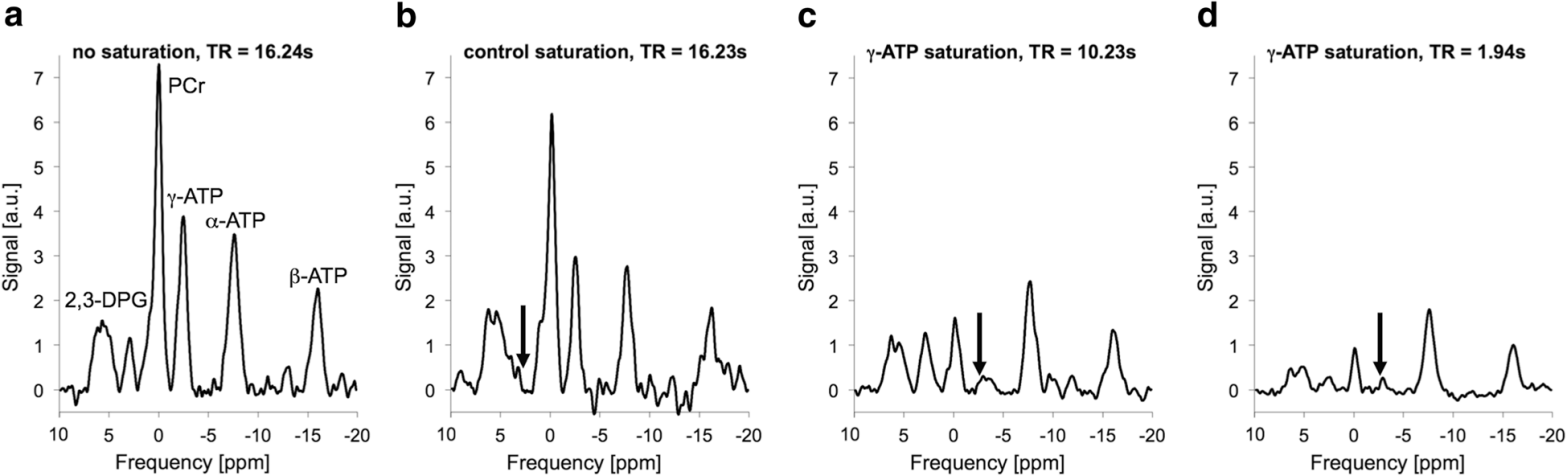
Example cardiac spectra acquired from the 6^th^ slice of 1DCSI setup illustrated in Fig. 1. **a** 9 min acquisition without any saturation used to determine M_0_ from the peak height of the PCr peak. This spectrum is also used to determine the saturation frequency of the γ-ATP peak in the heart. **b** 9 min acquisition with control saturation at the frequency indicated by the black arrow used to determine M_0_^control^. **c** 22 min acquisition with γ-ATP saturation (black arrow) and long TR used to determine M_0_` in the TwiST experiment, and M`(TR_long_) in the TRiST experiment. **d** This 9 min acquisition is only used in TRiST but not in TwiST. It is acquired with γ-ATP saturation (black arrow) and short TR and used to determine M`(TR_short_). TwiST uses acquisitions shown in **b**) and **c**); TRiST uses acquisitions shown in **b**), **c**), and **d**); Q-corrected TwiST uses acquisitions shown in **a**), **b**), and **c**); and Q-corrected TRiST uses acquisitions shown in **a**), **b**), **c**), and **d**)

Spectra from the anterior myocardium were analyzed as described in [7]. A semi-automatic tool (IDL 6.3, Exelis Visual Information Solutions, Boulder, Colorado) was used to measure PCr signals from peak heights after subtracting the baseline (Fig. 2). Peak height instead of area is used because signal ratios are determined from acquisitions with identical shim settings. User interactions were limited to the selection of cardiac slices (including quarter or half slice Fourier shift) and zero order phasing. Identical phasing is used for all four acquisitions. The baseline is determined automatically by averaging values around the minimum points of the peak of interest. T_1_` and M_0_` were determined from M`(TR_short_) and M`(TR_long_) using the dual-TR method [8]. To perform spill-over corrections, Q was determined for all cardiac spectra using Eq. [3]. Q values larger than one (due to SNR fluctuations) were set equal to 1.0.

T_1_^intrinsic^ and spill-over corrected T_1_^Q-intrinsic^ were calculated for each cardiac slice using Eq. [4] and [6], respectively, and averaged for each participant. The mean and SD of T_1_^intrinsic^ and T_1_^Q-intrinsic^ were determined for the healthy and the HF groups, and the two groups compared using an unpaired Student’s *t*-test. Uncorrected and Q-corrected intrinsic T_1_ values were compared with a paired Student’s *t*-test.

k_f_^TRiST^ and k_f_^TwiST^ were determined for all cardiac slices using Eq. [2] and [5], respectively. For k_f_^TwiST^, T_1_^intrinsic^ = 7.9 s was used. Spill-over corrected k_f_^Q-TRiST^ and k_f_^Q-TwiST^ were determined using Eq. [7] and [8]. All cardiac values were averaged for each participant. TRiST and TwiST k_f_ values were compared with and without Q-corrections using paired Student’s t-testing, linear regression, and Bland-Altman analysis. The mean and SD of Q-corrected k_f_^Q-TwiST^ were determined for the healthy and HF groups, and the two cohorts compared by unpaired Student’s t-testing. A *p* < 0.05 was considered significant for all statistical testing.

## Results

### Simulations

**Fig. 3:**
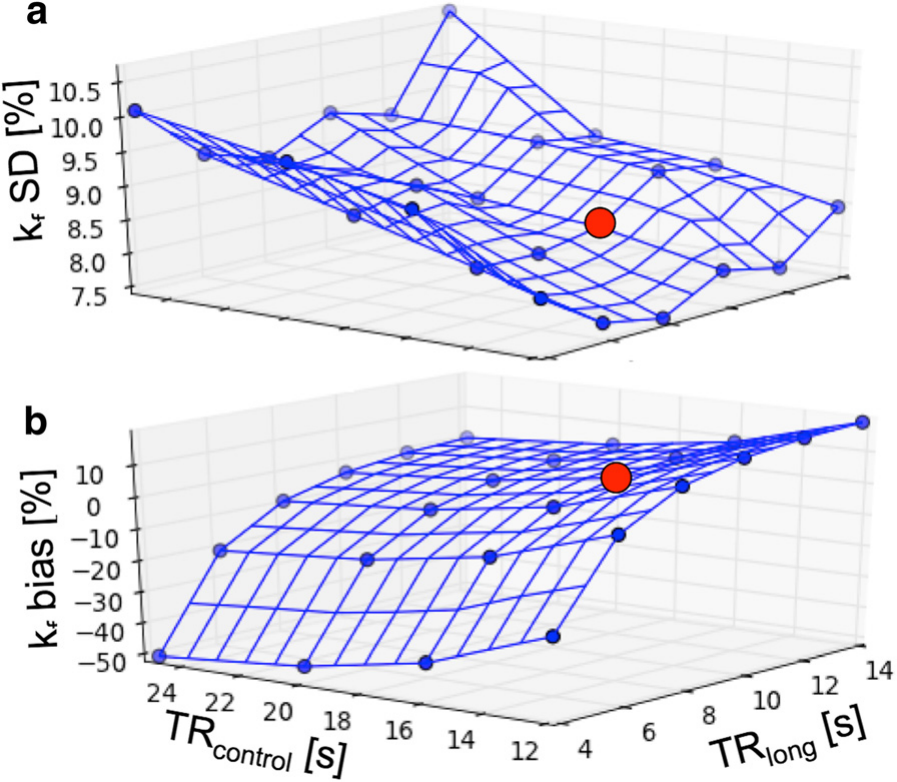
Results from Monte Carlo simulations of the TwiST experiment (without spill-over correction) over a range of TR_long_ (with γ-ATP saturated) and TR_control_ (control saturation), averaged over a range of k_f_ from 0.1-0.4 s^−1^ and T_1_^intrinsic^ from 5–9 s. Averages were chosen for a constant total study time of 30 min. **a** The relative percentage SD in k_f_ for average choices that led to the smallest error at each TR combination. **b** The average bias error in k_f_, corresponding to the same TR and average combinations as in a) and T_1_^intrinsic^ of 7 s. The red dot illustrates the TRs used in the human acquisitions in this study (TR_long_ = 10s and TR_control_ = 16 s)

Figure 3 shows the results of the Monte Carlo simulations without spill-over effects. Figure 3a and b can be compared directly to that of TRiST in Fig. 3 of [7]. The average SD (Fig. 3a) of TwiST is lower and varies less for different TRs than the TRiST result [7]. The expected noise-induced SD at the TRs used in the present study is 8.3 % compared to 13.4 % for TRiST [7]. Unlike TRiST, the bias error in TwiST (Fig. 3b) depends strongly on TR_long_. For TR_long_ < 8 s the negative bias error grows rapidly. The currently applied TR_long_ of 10 s is in the flat part of the graph and therefore a reasonable choice. In this regime, the bias error increases slightly when decreasing TR_control_, and TR_control_ =16 s remains a reasonable choice.

**Fig. 4:**
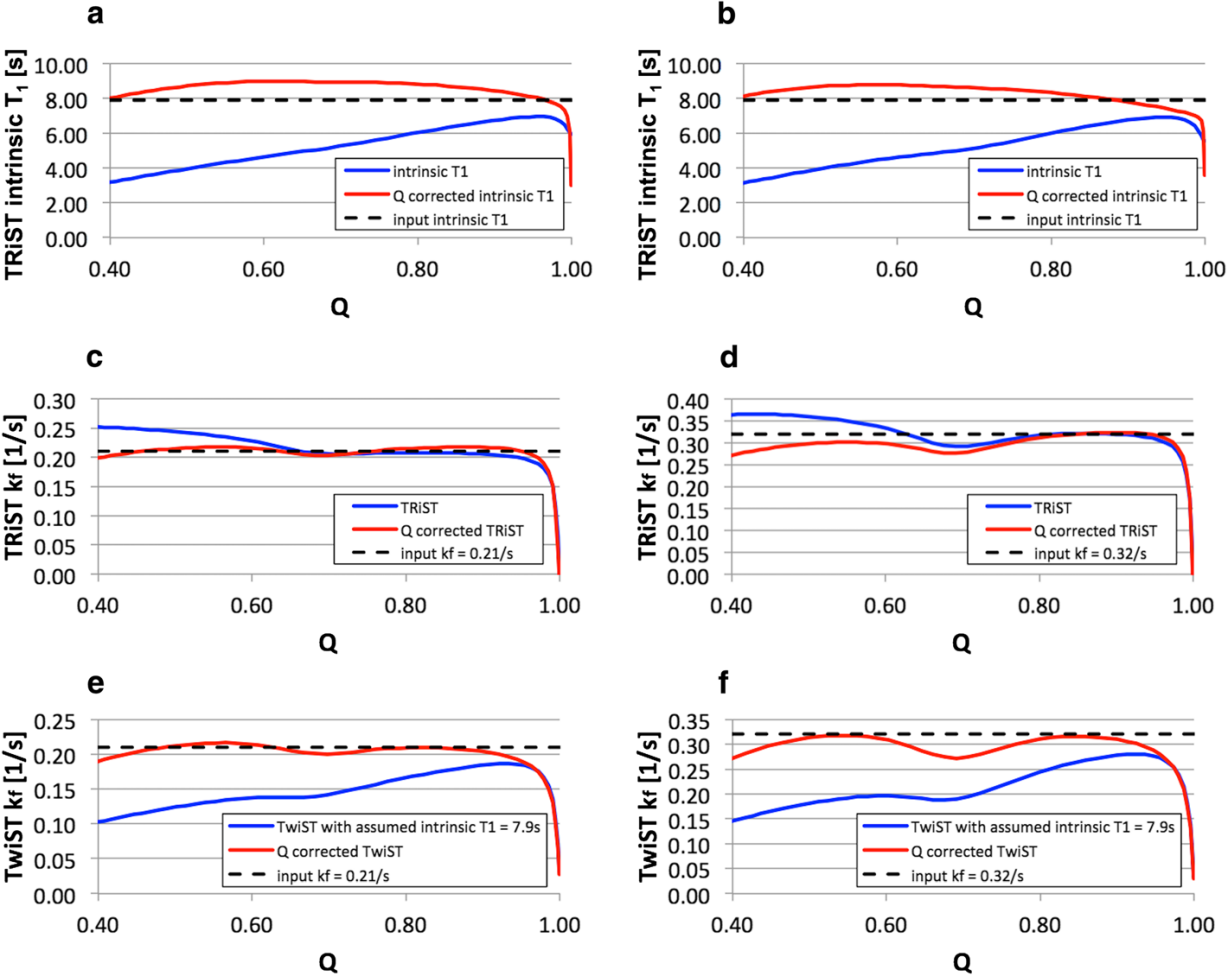
Spill-over effects as determined by Bloch equation simulations: T_1_^intrinsic^ (solid blue line) as determined with the TRiST method and spill-over corrected T_1_^Q-intrinsic^ (solid red line) are shown for **a**) k_f_ = 0.21 s^−1^ and for **b**) k_f_ = 0.32 s^−1^ as a function of the spill-over ratio Q. Input T_1_^intrinsic^ used in the simulation was 7.9 s (dotted black line). **c**) and **d**) show the pseudo-first-order rate constant k_f_ as determined with TRiST (solid blue line) and with Q-corrected TRiST (solid red line) for k_f_ = 0.21 s^−1^ and k_f_ = 0.32 s^−1^ (dotted black line), respectively. And k_f_ determined with TwiST (solid blue line) and Q-corrected TwiST (solid red line) is shown **e**) for k_f_ = 0.21 s^−1^ and **f**) for k_f_ = 0.32 s^−1^ (dotted black line). Note that the dip in T_1_^intrinsic^ and k_f_ for Q > 0.95 is caused by incomplete γ-ATP saturation, as the range in Q is induced by the variation of the DANTE saturation flip angles

The effects of spill-over as determined by the Bloch equation simulations are shown in Fig. 4. The T_1_^intrinsic^ (Fig. 4a and b, blue lines) determined in the presence of spillover underestimates the true value in proportion to the amount of spillover, as indexed by declining Q. The underestimation of T_1_^intrinsic^ is independent of k_f_ at 0.21 s^−1^ (Fig. 4a) vs. 0.32 s^−1^ (Fig. 4b). Figure 4c and d show the deviation in k_f_^TRiST^ (blue lines) from the true values (black, dashed lines) vs. Q for the TRiST method. The error stays within about 10 % for Q > 0.6 but increases with higher spill-over (Q < 0.6). For the TwiST method, k_f_^TwiST^ varies more strongly with Q (blue lines in Fig. 4e and f). At high levels of spill-over (Q ~ 0.4), k_f_^TwiST^ is underestimated by ~50 %, and a spill-over correction is required over most of the range.

**Fig. 5:**
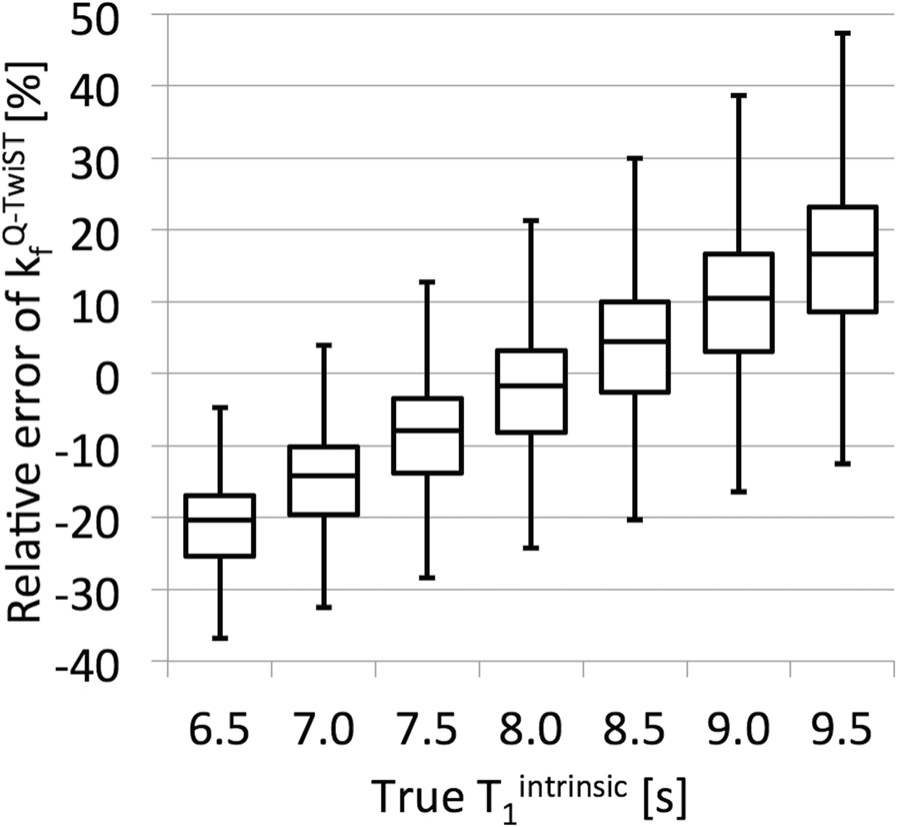
Boxplots of the simulated relative error of k_f_^Q-TwiST^ over the parameter range (Table 1) that was used to determine the coefficients for the spill-over corrected formulae plotted for the different intrinsic T_1_s used in the simulation

The coefficients of the spill-over corrected formulas for T_1_^Q-intrinsic^ (Eq. [6]), k_f_^Q-TRiST^ (Eq. [7]) and k_f_^Q-TwiST^ (Eq. [8]) are listed in Table 2. The calculated relative errors of T_1_^intrinsic^, k_f_^TRiST^ and k_f_^TwiST^ over the simulated range of parameters (Table 1) before and after spill-over correction are shown in Table 3. Spill-over correction removes the bias error and moderately reduces the error range, which includes the effect of a varying T_1_^intrinsic^ in accordance with Eq. 5. The relative error of k_f_^Q-TwiST^ for the same parameter range is plotted in Fig. 5 versus the T_1_^intrinsic^ used in the simulations. As expected, the bias error for T_1_^intrinsic^ outside of 8–8.5 s is proportional to the actual T_1_^intrinsic^. Applied to the simulations shown in Fig. 4, the spill-over correction (red lines) improves the determined intrinsic T_1_, (Fig. 4a and b) and TwiST k_f_ (Fig. 4e and f) for a wide range of Q, and TRiST k_f_ for Q < 0.6 (Fig. 4c and d).

**Table 2:** Coefficients for spill-over corrected T_1_^Q-intrinsic^(Eq. [6]), k_f_^Q-TRiST^ (Eq. [7]) and k_f_^Q-TwiST^ (Eq. [8]) were calculated for cardiac CK exchange measured with modulated DANTE saturation as described in [7] at 3 Tesla for a parameter range given in Table 1

Formula	Coefficients			
T_1_^Q-intrinsic^	a = 4.2803	b = −1.1344	c = −1.6610	d = 0.7862
k_f_^Q-TRiST^	f = 0.0052	g = −0.8730	h = 27.5332	k = −6.0647
k_f_^Q-TwiST^	l = −0.2013	m = −1.0305	n = −1.6113

**Table 3:** Simulated relative errors of T_1_^intrinsic^, k_f_^TRiST^ and k_f_^TwiST^ before and after Q spill-over correction determined over the range of parameters (Table 1) used to determine the coefficients for the spill-over corrected formulae (Table 2), including the effect of a varying T_1_^intrinsic^ per Eq. [5]

	Relative error [%] (mean ± SD)	Relative error [%] range (min, max)
T_1_^intrinsic^ (Eq. 4)	−37.0 ± 16.4	(−66.2, −9.9)
T_1_^Q-intrinsic^ (Eq. 6)	0.8 ± 7.9	(−23.2, 16.2)
k_f_^TRiST^ (Eq. 2)	12.0 ± 12.8	(−8.8, 71.8)
k_f_^Q-TRiST^ (Eq. 7)	−1.1 ± 10.3	(−35.8, 40.1)
k_f_^TwiST^ (Eq. 5)	−27.6 ± 17.7	(−60.6, 27.6)
k_f_^Q-TwiST^ (Eq. 8)	−2.3 ± 14.9	(−36.8, 47.3)

### Experiments

The total acquisition time including repositioning and scout MRI at the beginning of each study to optimally position the coil for cardiac TRiST was 84 ± 10 min (mean ± SD) for all participants. This included the 9 min to acquire the unsaturated M_0_ data set with no saturation used for the Q corrections and metabolite quantification. The duration of the three TRiST acquisitions was 40 ± 1 min. Omitting the M`(TR_short_) acquisition, which is no longer needed for TwiST, reduces the effective acquisition time by 9 ± 1 min, for a 23 % efficiency improvement for TwiST.

**Fig. 6:**
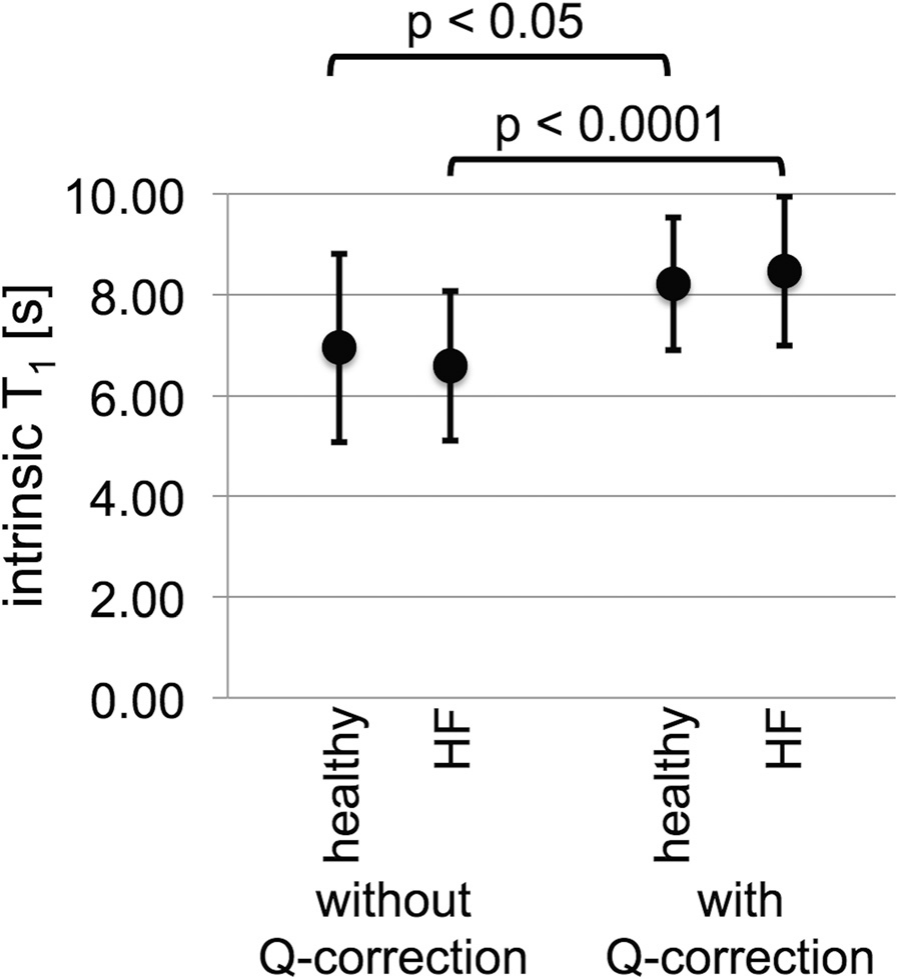
Cardiac PCr T_1_^intrinsic^ and Q-corrected T_1_^Q-intrinsic^ determined with the TRiST method for healthy (*n* = 12) and for HF patients (*n* = 17), shown are mean (dot) and standard deviation (error bars). The intrinsic T_1_ between the two groups is not significantly different both without and with Q-correction, *p* = 0.57 and *p* = 0.63, respectively. Q-correction leads to a significant increase of the intrinsic T_1_ for both the healthy and HF groups with *p* < 0.05 and *p* < 0.0001, respectively

Cardiac PCr T_1_^intrinsic^ are shown in Fig. 6, both with and without Q-correction. T_1_^intrinsic^ with and without Q-correction does not differ significantly between patients and healthy controls. However, spill-over correction significantly increases the intrinsic T_1_. With Q-correction, T_1_^Q-intrinsic^ = 8.2 ± 1.3 s in healthy subjects vs. 8.5 ± 1.5 s in HF (*p* = 0.6). The average spill-over corrected T_1_^Q-intrinsic^ in all participants is 8.4 ± 1.4 s. The Q values were the same in both groups at 0.84 ± 0.13 in healthy subjects and 0.85 ± 0.11 in HF (*p* = 0.8).

**Fig. 7:**
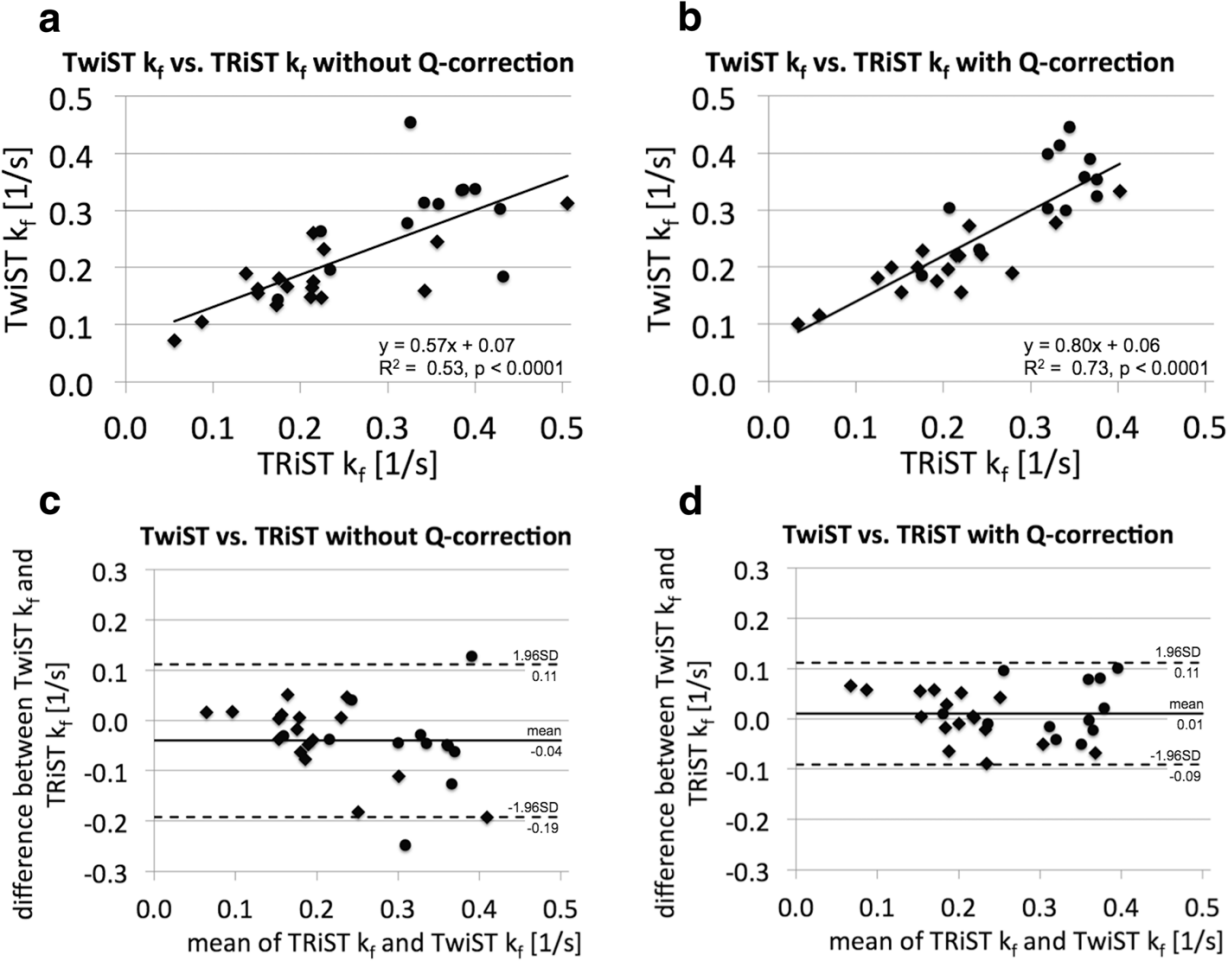
**a**, **b** Linear correlation and (**c**, **d**) Bland-Altman plots for TRiST and TwiST cardiac CK pseudo-first-order rate constant k_f_ determined (**a**, **c**) without and (**b**, **c**) with spill-over Q-correction. Data from all 29 participants are shown, dots for healthy participants and diamonds for HF patients

TwiST k_f_ measurements are compared to the previously validated TRiST k_f_ in Fig. 7. Linear regression (Fig. 7a and b) reveals a significant correlation between k_f_^TRiST^ and k_f_^TwiST^ calculated both without (R^2^ = 0.53) and with (R^2^ = 0.73) Q-corrections (*p* < 0.0001 for both). Corresponding Bland-Altman plots in Fig 7c and d show that the Q-correction reduces scatter.

**Fig. 8:**
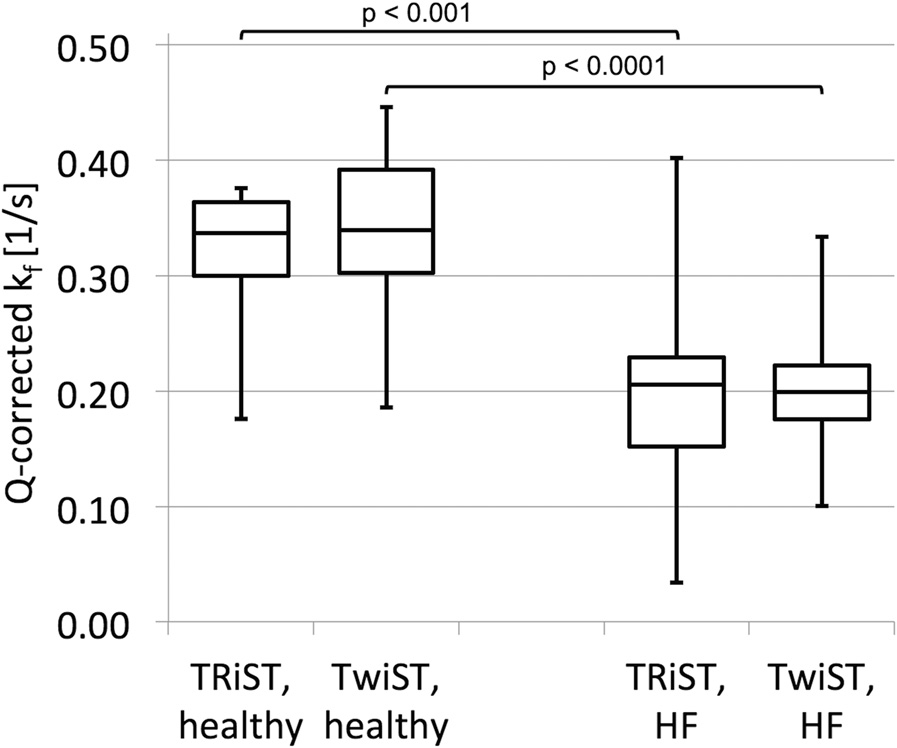
Boxplot of cardiac CK pseudo-first-order rate constant k_f_ determined with spill-over Q-corrected TRiST and TwiST for healthy and heart failure patients. k_f_ measured with Q-corrected TRiST and TwiST are the same for both healthy (*p* = 0.22) and HF patients (*p* = 0.79). Cardiac CK k_f_ in HF is significantly reduced compared to that in healthy subjects

Figure 8 presents box plots for k_f_ determined with spill-over Q-corrected TRiST and TwiST methods for healthy and HF patients measured at 3 T. K_f_^Q-TwiST^ are the same as k_f_^Q-TRiST^ values for both healthy subjects (*p* = 0.2) and HF patients (*p* = 0.8). The TwiST CK rate constant in HF patients was 0.20 ± 0.06 s^−1^ (mean ± SD), significantly lower than that in the healthy group at 0.33 ± 0.08 s^−1^ (*p* = 0.00001). Results of the previously published TRiST analysis without Q-correction are compared to the ones with Q-correction in an additional figure [see Additional file 1].

## Discussion

We present a new, faster method called TwiST for measuring the forward CK rate-constant in human heart. The method is validated by Bloch equation analysis and by comparison with the previously validated TRiST method in ^31^P MRS studies of healthy and failing human hearts performed at 3 T. The TwiST method is faster than the TRiST method, requiring one less acquisition and saving 9 min from the present protocol, or a 23 % efficiency improvement vs. the three TRiST acquisitions. The number of acquisitions required for measuring CK reaction rates has thus now been reduced from four [3] or three [7] to two, resulting in proportionate improvements in efficiency for the ST portion of the protocol. Although the timesaving is not large relative to the entire protocol, it does shorten a long exam, making it more tolerable for patients with cardiovascular disease without introducing significant error.

This study also presented the first 3 T measurements of cardiac CK kinetics in patients with heart failure. The results show significant reductions in cardiac CK reaction-rates that are in quantitative agreement (both mean values and errors) with prior measurements obtained at 1.5 T, where k_f_ was 0.21 ± 0.07 s^−1^ in HF patients compared to 0.32 ± 0.07 s^−1^ in healthy subjects [3]. The new measurements obtained by both TRiST and TwiST methods and at a different field strength of 3 T, provide further independent evidence that CK energy supply is reduced in the failing human heart. A paired comparison of data acquired by the different methods at 1.5 T and 3 T from the same subjects was not performed here, as the original 1.5 T scanner is no longer available. Such studies could help elucidate whether the residual scatter has biologic or instrumental origins.

The Monte Carlo simulations show that the expected scatter for a given SNR decreases to 8.3 % in TwiST measurements compared to 13.4 % with TRiST. This is because in TwiST a T_1_^intrinsic^, or a range of T_1_^intrinsic^ for the Q-corrected TwiST, is assumed instead of measuring T_1_`. In TRiST, T_1_` is determined from two measurements: M`(TR_long_) and M`(TR_short_). Compared to TRiST, TwiST does not measure M`(TR_short_). M`(TR_short_) is the acquisition with the lowest PCr signal in TRiST because of the short TR and its chemical exchange with the saturated γ-ATP. The combination of low signal for M`(TR_short_) and the inherently low SNR in clinical cardiac ^31^P MRS settings, makes the determination of T_1_` critical to the accuracy of TRiST k_f_ determinations.

Bloch equation simulations showed that T_1_^intrinsic^ measured with the TRiST sequence underestimates the true value to an extent that depends strongly on the spill-over ratio Q. This can confound its determination. For example, assuming an input T_1_^intrinsic^ of 7.9 s, the simulations predict apparent T_1_^intrinsic^ values of 4 to 7 s as Q varies from 0.5 to 1 (Fig. 4a and b). In the present study, the measured T_1_^intrinsic^ varied from 3.4 to 10.7 s. We therefore assumed a range in the actual intrinsic T_1_ from 6.5 to 9.5 s for computing the Q-corrected T_1_^intrinsic^ and TRiST/TwiST k_f_ formulae. For the Monte Carlo simulations without spill-over corrections, T_1_^intrinsic^ = 7 s was chosen to be consistent with simulations performed in [7], and to enable a comparison of the findings. In the present study, there were no significant differences in T_1_^intrinsic^ between healthy subjects and HF patients, whether calculated with or without Q-corrections (Fig. 6). This suggests that the same T_1_^intrinsic^ can be assumed for TwiST studies of k_f_ in HF patients and healthy subjects. The overall average value pooling the HF patients and healthy subjects was 8.4 ± 1.4 s. That T_1_^intrinsic^ is the same, is also consistent with the notion that T_1_^intrinsic^ for PCr is a measure of T_1_ independent of any exchange effects or differences therein in healthy and HF populations.

The proposed formula for spill-over corrected TwiST, Eq. [8], does not explicitly include T_1_^intrinsic^. Nevertheless, the coefficients in Table 2 depend on the range of T_1_^intrinsic^ assumed for their determination. For Q = 1, Eq. 8 can be transformed into an equation similar to Eq. 5,

9 $$ {k}_f^{Q- TwiST}\left(Q=1\right)=\frac{1}{8.13}\left(\frac{M_0^{control}}{M\hbox{'}\left(T{R}_{long}\right)}-1.03\right), $$

with an equivalent T_1_^intrinsic^ of 8.13 s very close to the value measured in this study. Hence, T_1_^intrinsic^ is absorbed into coefficients *l* and *n* of Eq. 8. The effect of varying T_1_^intrinsic^ on k_f_^Q-TwiST^ can be determined from Fig. 5. Apart from this, the coefficients in Table 2 for the spill-over corrected formulae for T_1_^Q-intrinsic^, k_f_^Q-TRiST^ and k_f_^Q-TwiST^ are only applicable for data acquired with the sequence parameters used in the present study to measure cardiac CK exchange rates at 3 T with the expected parameter range as given in Table 1. Deviations would in general require determination of a new set of coefficients based on adapted simulations.

The Bloch equation simulation results in Fig. 4 suggest that for Q > 0.95 spill-over effects lead to a dip of T_1_^intrinsic^ and both the TRiST and TwiST k_f_ measurements. Q values larger than 0.95 only occur for very low saturation power and the dip in T_1_^intrinsic^ and TRiST/TwiST k_f_ for Q values larger than 0.95 is caused by incomplete γ-ATP saturation. In practice, Q values larger than 0.95 can occur because of low saturation power (leading to both reduced spill-over saturation of PCr and incomplete γ-ATP resonance saturation) or because of noise in the acquired spectra. The former can be assessed in the spectra by noting any residual γ-ATP resonance. We attributed Q > 1 to noise and rounded Q to 1 in the Q-corrected formulae. Based on Eq. [3], the error in *Q* is the root of the sum of the squared errors in *M*_*0*_ and *M*_*0*_^*control*^. In the determination of the Q-corrected formulae the range of saturation power was limited to keep Q below 0.96 to ensure that the dip was not included in the fitting coefficients.

Recently, Bashir et al. presented a time-dependent ST approach to measure CK k_f_ values in the human heart at 3 Tesla [24]. Their reported k_f_ = 0.32 ± 0.05 s^−1^ agrees well with k_f_ values of the present work, whereas their PCr T_1_^intrinsic^ = 7.36 ± 1.79 s is somewhat smaller than the Q-corrected T_1_^Q-intrinsic^ = 8.4 ± 1.4 s presented here. Xiong et al. published a very fast ST method applied to *in vivo* swine hearts at ultra-high field strength (4.7 T - 9.4 T) [25, 26]. Their fastest 1D CSI localized T_1_^nom^ method acquires the ST protocols in less than 14 min. This compares to ~40 min for our Q-corrected TwiST protocol that includes a third acquisition for measuring metabolite concentrations and Q. The T_1_^nom^ method has yet to be translated to human heart studies or combined with concentration measurements. Also it is not compensated for spillover which may be more problematic at lower fields where chemical shift dispersions are proportionately smaller.

### Limitations

Test re-test reproducibility of these methods remains to be studied in the future. The transmit/receive coil and pulse sequences used in this study have been specially designed and built by our research team for cardiac ^31^P MRS.

## Conclusions

In conclusion, the spill-over Q-corrected TwiST method can be used to measure the CK pseudo-first-order rate-constant k_f_ in the human heart at 3 Tesla with one fewer acquisition compared to the previously presented TRiST method. Instead, a range of PCr T_1_^intrinsic^ is assumed. It is shown that T_1_^intrinsic^ is the same in healthy subjects and in heart failure patients. The values of k_f_ measured with Q-corrected TwiST closely agree with earlier measurements at 1.5 Tesla, and demonstrate a significant reduction in failing, compared to healthy hearts.

## Additional file

Additional file 1:
**Boxplot of cardiac CK pseudo-first-order rate constant k**_**f**_ **determined with TRiST, spill-over Q-corrected TRiST, and spill-over Q-corrected TwiST for healthy and heart failure patients.** k_f_ measured with TRiST, with Q-corrected TRiST, and with Q-corrected TwiST are the same in both healthy and HF patients. Cardiac CK k_f_ in HF is significantly reduced compared to that in normal subjects. (PDF 60 kb)

## Abbreviations

1D CSI: One-dimensional chemical shift imaging
^31^P: Phosphorus
ATP: Adenosine triphosphate
CK: Creatine kinase
DANTE: Delays alternating with nutations for tailored excitation
HF: Heart failure
PCr: Creatine phosphate
Q: Spill over ratio, see equation 3
SD: Standard deviation
ST: Saturation transfer
TRiST: Triple Repetition time Saturation Transfer
TwiST: Two Repetition time Saturation Transfer
