# Supplementary material for: Two repetition time saturation transfer (TwiST) with spill-over correction to measure creatine kinase reaction rates in human hearts
Source: J Cardiovasc Magn Reson. 2015 Aug 8;17(1):70. doi: 10.1186/s12968-015-0175-4 (PMC4529717; doi:10.1186/s12968-015-0175-4)
Supplement: Additional file 1: — Boxplot of cardiac CK pseudo-first-order rate constant k f determined with TRiST, spill-over Q-corrected TRiST, and spill-over Q-corrected TwiST for healthy and heart failure patients. kf measured with TRiST, with Q-corrected TRiST, and with Q-corrected TwiST are the same in both healthy and HF patients. Cardiac CK kf in HF is significantly reduced compared to that in normal subjects. (PDF 60 kb) [file 12968_2015_175_MOESM1_ESM.pdf]

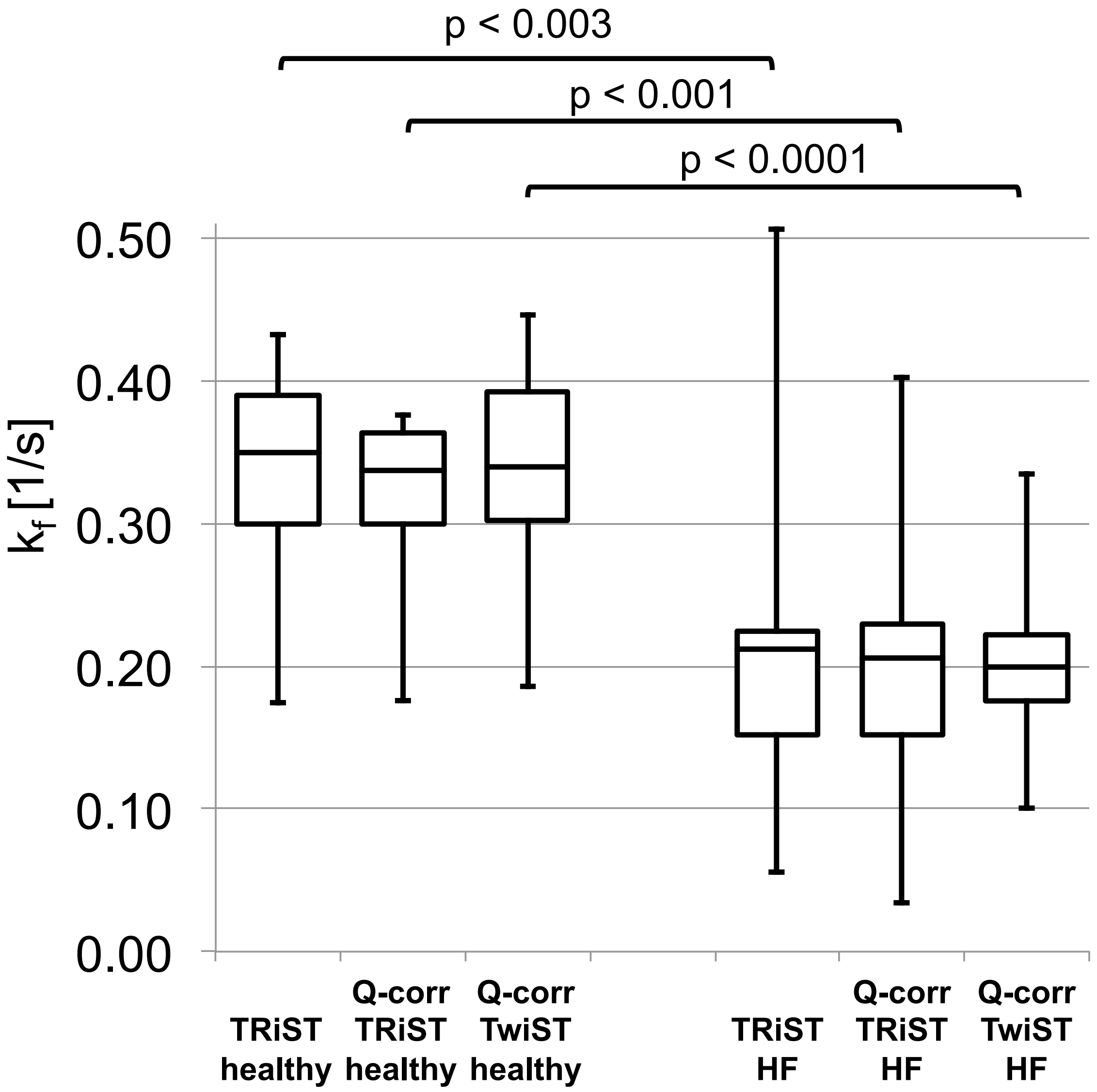

Additional Figure: Boxplot of cardiac CK pseudo-first-order rate constant k_f determined with TRiST, spill-over Q-corrected TRiST, and spill-over Q-corrected TwiST for healthy and heart failure patients. k_f measured with TRiST, with Q-corrected TRiST, and with Q-corrected TwiST are the same in both healthy and HF patients. Cardiac CK k_f in HF is significantly reduced compared to that in normal subjects.
